# Evaluation of Subcutaneous and Intermuscular Adipose Tissues by Application of Pattern Recognition and Neural Networks to Ultrasonic Data: A Model Study †

**DOI:** 10.3390/bioengineering12121373

**Published:** 2025-12-17

**Authors:** Alexey Tatarinov, Aleksandrs Sisojevs, Vladislavs Agarkovs, Jegors Lukjanovs

**Affiliations:** Institute of Electronics and Computer Science, LV-1006 Riga, Latvia; aleksandrs.sisojevs@edi.lv (A.S.);

**Keywords:** intermuscular and subcutaneous adipose tissues, ultrasonic measurements, pattern recognition, artificial neural networks, tissue models

## Abstract

Distinguishing subcutaneous adipose tissue (SAT) from intermuscular adipose tissue (IMAT) is clinically important because IMAT infiltration is strongly associated with age-related functional decline, sarcopenia, diabetes, cardiovascular disease, and obesity. Current assessments rely on MRI or CT, which are stationary, costly, and labor-intensive. Portable ultrasound-based solutions could enable broader, proactive screening. This model study investigated the feasibility of differentially assessing SAT and IMAT using features extracted from propagating ultrasound signals. Twenty-five phantoms were constructed using gelatin as a muscle-mimicking matrix and oil as the SAT and IMAT compartments, arranged to provide gradual variations in fat fractions ranging from 0% to 50%. Ultrasound measurements were collected at 0.8 MHz and 2.2 MHz, and multiple evaluation criteria were computed, including ultrasound velocity and parameters derived from the signal intensity. Classification domains were then generated from intersecting decision rules associated with these criteria. In parallel, artificial neural networks (ANN/LSTM) were trained and tested on identical phantom subsets to evaluate data-driven classification performance. Both the rule-based and ANN/LSTM approaches achieved diagnostically meaningful separation of SAT and IMAT. The aim of this work was to perform an experimental proof-of-concept study on idealized tissue models to demonstrate that ultrasound measurements can reliably differentiate SAT and IMAT, supporting the development of future screening devices.

## 1. Introduction

Subcutaneous adipose tissue (SAT) and intermuscular adipose tissue (IMAT) are two distinct fat depots in human limbs with markedly different biological, functional, and clinical implications. SAT is the widely distributed subcutaneous fat layer, composed of adipocytes located just beneath the skin, surrounding muscles externally. IMAT is fatty infiltration located between and within muscle groups in the perimuscular and intermuscular compartments. This includes visible lipid storage in adipocytes located between muscle fibers and between muscle groups [1]. Although both depots contribute to whole-body adiposity, their physiology and pathological significance differ substantially.

While total adiposity is a strong predictor of cardiometabolic risk, differentiating SAT from IMAT is critical in musculoskeletal, metabolic, and geriatric medicine. Excess SAT in the limbs primarily reflects energy storage and does not directly impair muscle contractility. In contrast, IMAT compromises muscle strength per unit area, reduces insulin sensitivity, and exacerbates mobility decline [2,3]. Clinical studies show that IMAT, and not SAT, is the stronger predictor of falls, frailty, and impaired rehabilitation outcomes after orthopedic surgery [4,5]. Thus, precise assessment of both compartments allows for improved stratification in osteosarcopenia, sarcopenic obesity, diabetes, and neuromuscular disorders.

SAT thickness in human lower limbs, such as the thigh and calf, typically ranges from about 4 to 20 mm depending on sex, body mass index (BMI), and location, with the anterior thigh often thicker than the lateral thigh or calf [6]. Measurements obtained by different techniques show large inter-subject variability. In normal-weight adults, SAT in the thigh and calf regions usually varies between 4 and 12 mm, whereas in overweight and obese individuals, it can reach up to 40 mm or more [7]. Volumetric IMAT values, reported as a percentage of the muscle compartment in thigh muscles in normal to pathological cohorts, range from about 1 percent to more than 30 to 35 percent [8].

There are several clinical and pathophysiological reasons for differential evaluation of SAT and IMAT. From a metabolic perspective, IMAT is strongly associated with insulin resistance, type-2 diabetes, and systemic metabolic dysfunction, even after controlling for total fat. IMAT and intramyocellular lipid correlate with impaired glucose uptake and local inflammatory signaling [9]. SAT, by contrast, is metabolically “safer” in many contexts. In older adults, elevated IMAT infiltration correlates with reduced strength, poorer mobility, worse surgical outcomes, and higher frailty associated with sarcopenia, myosteatosis, and sarcopenic obesity [4,10]. Differential measurement helps identify sarcopenic obesity and stratify patients into risk groups. Infiltration of fat within and between muscles, known as myosteatosis, alters muscle quality and has local mechanical and functional consequences, particularly impacting on contractile efficiency, while also promoting fibrosis [11]. Therefore, IMAT estimation is relevant for rehabilitation and orthopedic planning. Separate knowledge of IMAT and SAT is important in disease-specific contexts such as obesity, type-2 diabetes mellitus, chronic kidney disease, chronic inflammatory states, and muscle atrophy after disuse or immobilization, and it helps to understand disease mechanisms and identify potential therapeutic targets. It is also recognized that exercise preferentially reduces IMAT [8]. Some studies have demonstrated an independent association of IMAT deposition in the thigh with cardiometabolic risk factors, including total blood cholesterol, low-density lipoprotein (LDL), and triglycerides, as well as decreased insulin sensitivity. The SAT-to-IMAT ratio is associated with serum adiponectin levels and may serve as an indicator of individual cardiovascular risk [12].

**Figure 1:**
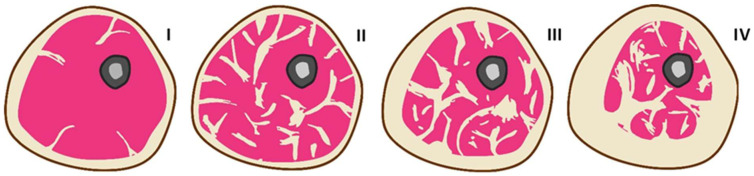
Schematic representation of SAT and IMAT in cross-section of a human limb under various typical conditions: I—healthy; II—type-2 diabetes; III and IV—progressing stages of obesity.

Figure 1 schematically shows cross-sections of a hypothetical human limb, demonstrating various SAT-to-IMAT ratios under different conditions, based on a series of literary sources [13,14,15,16] that provide corresponding tomographic images. In a healthy, normal-weight human limb, SAT typically constitutes the predominant fat compartment, while IMAT is minimal, resulting in a high SAT-to-IMAT ratio (I). In a type-2 diabetes patient with significant IMAT infiltration, the SAT-to-IMAT ratio is reduced due to a relative increase in IMAT with normal or even reduced SAT, reflecting abnormal fat redistribution within the limb (II). During the progression of obesity, SAT initially expands, predominantly maintaining a high SAT-to-IMAT ratio, but with advancing obesity and metabolic dysfunction, IMAT gradually increases, leading to a decline in the SAT-to-IMAT ratio as fat increasingly infiltrates muscle compartments (III–IV).

Non-invasive techniques for measuring SAT and IMAT in human limbs rely on accurate depot-specific quantification using imaging and either a tissue-specific signal, as in magnetic resonance imaging proton density fat fraction (MRI PDFF), an attenuation signature, as in CT-based Hounsfield unit segmentation (CT HU), or validated surrogates, as in ultrasonography. MRI PDFF directly estimates fat fraction and can biochemically separate intra- and extramyocellular lipids in a localized voxel. Its high soft tissue contrast allows for fascia-aware segmentation of SAT and IMAT and enables longitudinal quantification [16,17,18]. However, because of concerns regarding cost, limited access, and the stationary nature of scanners, MRI is mostly restricted to research settings where precise depot separation and quantitative tracking are required. In CT HU-based segmentation, anatomical cross-sectional areas and volumes that differ in radiological density are measured [19]. Fat is identified by low Hounsfield unit values, where about 190 HU is typical for fat and about 30 HU is typical for muscle. An advantage of CT is the possibility to quantify muscle density, also termed muscle attenuation or muscle radiation attenuation, which depends linearly on muscle fat content [20]. The high spatial resolution of CT permits retrospective opportunistic analyses from clinical scans, but the modality is limited by ionizing radiation, stationarity, and availability.

Ultrasonography, or clinical ultrasound, which explores the association between skeletal muscle echogenicity and its physical properties, presents an opportunity for use as a screening tool [6,21]. It is mainly applied to quantify SAT thickness, whereas reliable prediction of IMAT remains problematic, despite the fact that muscle echo intensity and texture have been identified as surrogate markers for muscle fat.

There is a sound physical and physiological rationale for exploring single channels through transmission ultrasound to estimate SAT and IMAT separately, although the method is still experimental and faces practical challenges. The physical basis is the strong dependence of the acoustic properties of tissues on fat content [22,23,24]. Acoustic impedance, ultrasound velocity (speed of sound), and attenuation differ systematically between lean and adipose tissues. The speed of sound in lean muscle tissue ranges from about 1540 to 1580 m/s, whereas in adipose tissue, it ranges from about 1440 to 1480 m/s. Attenuation is about 0.3 to 0.7 dB/cm per MHz in lean tissue and about 0.6 to 1.0 dB/cm per MHz in adipose tissue. Thus, systematic accumulation of fat in tissue should result in a linearly decreasing trend in sound velocity and an increasing trend in attenuation [25].

These differences produce layer-specific acoustic signatures in through-transmission measurements and cause characteristic refraction, attenuation, and frequency dispersion, which, in principle, can be inverted to estimate the thickness and composition of tissue layers. By analyzing the through-transmitted signal in a broadband frequency range, arrival time and the phase velocity can be used to estimate apparent bulk velocity, while amplitude decay and spectral slope can be used to estimate attenuation. Inversion with a layered model can then be used to estimate SAT thickness (layer 1) and IMAT-induced changes in the muscle layer (layer 2). This inversion is analogous to geophysical layer property estimation or ultrasonic inversion for bone thickness and porosity [26]. If such a quantitative ultrasound solution is implemented, it has the potential to become an inexpensive and portable alternative to MRI and CT.

Approaches of pattern recognition for the evaluation of factors of interest based on ultrasonic parameters as decision rules have been developed previously for different physical objects and application tasks. For example, the possibility of determining the depth and the degree of deterioration of the surface layer of concrete by the combined use of ultrasonic surface waves at different frequencies was proven by implementing a mathematical approach of pattern recognition using statistical criteria and neural networks [27,28]. The universality of this approach was shown for testing different materials with differentiation of factors of interest [29,30]. The method for estimating cortical thickness and intracortical porosity in compact bone models of osteoporosis relied on two complementary sources of information: statistical features derived from the ultrasonic signals themselves and physical parameters of ultrasound propagation obtained from the spatiotemporal waveform profiles [31,32]. Artificial neural network (ANN) methods are also used in biomedicine to analyze temporal signals (including ultrasound). For example, the bidirectional long short-term memory (BLSTM) module was used to analyze audio signals [33]. Respiratory pattern monitoring was performed based on temporal signal analysis using recurrent neural networks (RNNs) [34].

The aim of the present study was to demonstrate the potential for differential assessment of SAT and IMAT in simple muscle models by applying pattern recognition methods and neural network analysis to data derived from ultrasound signals obtained in through-transmission.

## 2. Materials and Methods

### 2.1. Muscle Mimicking Phantoms with SAT and IMAT Variation

**Figure 2:**
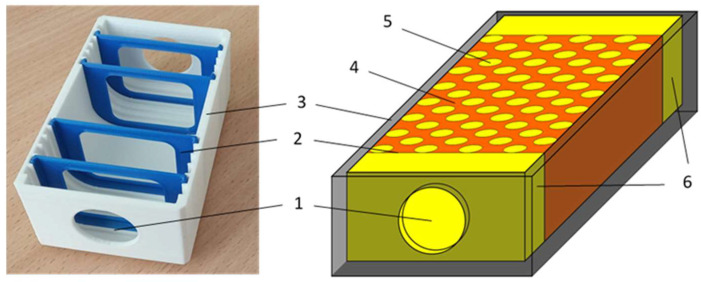
Ultrasonic tissue phantom: 1—acoustic window; 2—acoustically transparent membranes; 3—3D-printed mold; 4—“muscle tissue” section; 5—periodic oil-filled holes mimicking IMAT; 6—oil-filled sections mimicking SAT.

**Figure 3:**
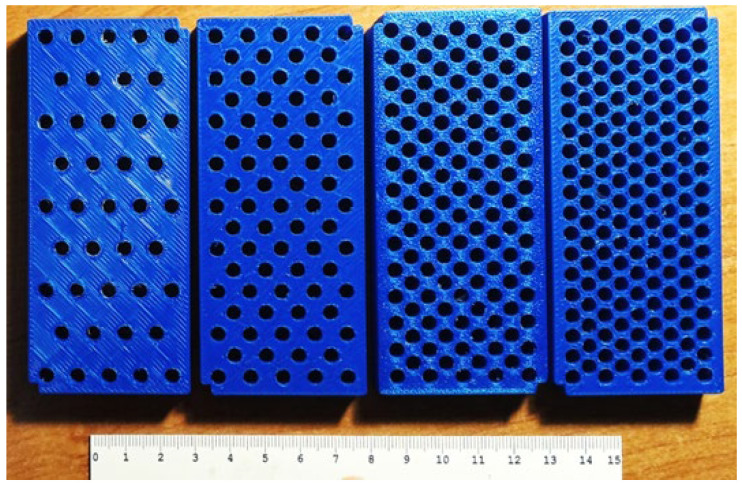
Figure showing 3D-printed dies with regular holes for creating IMAT gradations.

Ultrasound tissue phantoms were designed to simulate the combined effects of SAT and IMAT in a simplified muscle model over ranges close to physiological conditions associated with different clinical states. The phantom shown in Figure 2 consisted of a three-dimensionally printed plastic mold of 80 × 40 × 30 mm^3^ with fixed grooves for placing acoustically transparent partitions that separated the SAT sections. On the opposite edges of the long side, windows were covered with acoustically transparent membranes for the attachment of ultrasonic transducers used for through-transmission measurements. Symmetric SAT sections were placed at the opposite edges of the phantom, and the central region represented a “muscle tissue” section with simulated IMAT inclusions. SAT content was varied by changing the thickness of the symmetric SAT sections at the edges. IMAT content was varied by cylindrical inclusions of 5 mm diameter arranged in a staggered pattern in the central muscular region. The IMAT inclusions (holes) were formed using specially printed inclusion setting dies (Figure 3).

Because the ultrasonic measurements were carried out at room temperature (21 to 23 °C), the materials used to simulate the muscle tissue matrix and adipose tissue (SAT and IMAT) were selected so that their acoustic properties at room temperature would approximately match those of lean muscle and fat at body temperature (about 37 °C). The muscle tissue matrix was mimicked by a stiffened aqueous solution containing 20% animal gelatin. This gelatin-based gel has ultrasound velocities in the range 1570 to 1580 m/s at room temperature [35], which is close to ultrasound velocities measured in non-obese human muscle in vivo [36]. The fat content for both SAT and IMAT inclusions was mimicked by extra virgin olive oil, with an ultrasound velocity at room temperature of 1453 ± 2 m/s [37]. This value closely matches the typical ultrasound velocity of human adipose tissue, about 1450 m/s at 37 °C [24,38]. In this model, anisotropy of acoustic properties due to the presence and orientation of muscle fibers in real muscle was not taken into account. Both SAT and IMAT contents were gradually varied from 0 to 50%, with a step of 12.5%. In this design, SAT gradations were determined by the thickness of the SAT layer relative to the entire volume of the phantom (Figure 2), whereas IMAT gradations were related to the remaining part of the muscle component, or gelatin matrix, defined by the concentration of inner holes (Figure 3). The set included a grid of 25 phantoms with five levels of SAT and five levels of IMAT that varied independently, representing 25 possible SAT and IMAT combinations.

### 2.2. Ultrasonic Measurements

**Figure 4:**
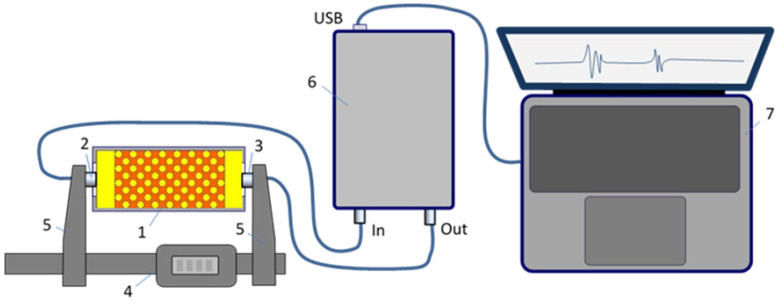
Experimental setup: 1—muscle tissue phantom; 2—ultrasound-receiving transducer; 3—ultrasound-emitting transducer; 4—digital caliper; 5—reinforced consoles; 6—ultrasonic acquisition unit; 7—laptop PC.

Ultrasonic signals were obtained by testing the phantom in through-transmission mode using a pair of transmitting and receiving transducers rigidly mounted on the opposite arms of a digital caliper, ensuring parallelism and centering of the transducers. The experimental setup is shown in Figure 4.

Acquisition of ultrasonic signals was controlled by a custom circuit based on a Cyclone IV EP4CE22E22C8 (Altera, San Jose, CA, USA) field programmable logic array (FPGA), which communicated with a computer via a USB 3.0 interface chip FIFO FT600. The FPGA controlled a high-voltage pulsar HV7360 (Microchip, Chandler, AZ, USA) that generated ultrasonic tone pulses with output voltages up to 200 V peak-to-peak. Received ultrasonic signals were amplified by a voltage gain amplifier AD8367ARUZ (Analog Devices, Wilmington, MA, USA), with gain up to 45 dB, and digitized by an LTC2250 analog-to-digital converter (Analog Devices), a 10-bit, low-noise, 125 Msps device designed for digitizing high-frequency signals with a wide dynamic range. High- and low-voltage rails in the circuit were provided by an LT8331 (Analog Devices) DC to DC converter. The FPGA processing core managed transmission and reception data streams in FIFO buffer memory registers and additional buffers for digitized received signals and transmitted waveforms.

**Figure 5:**
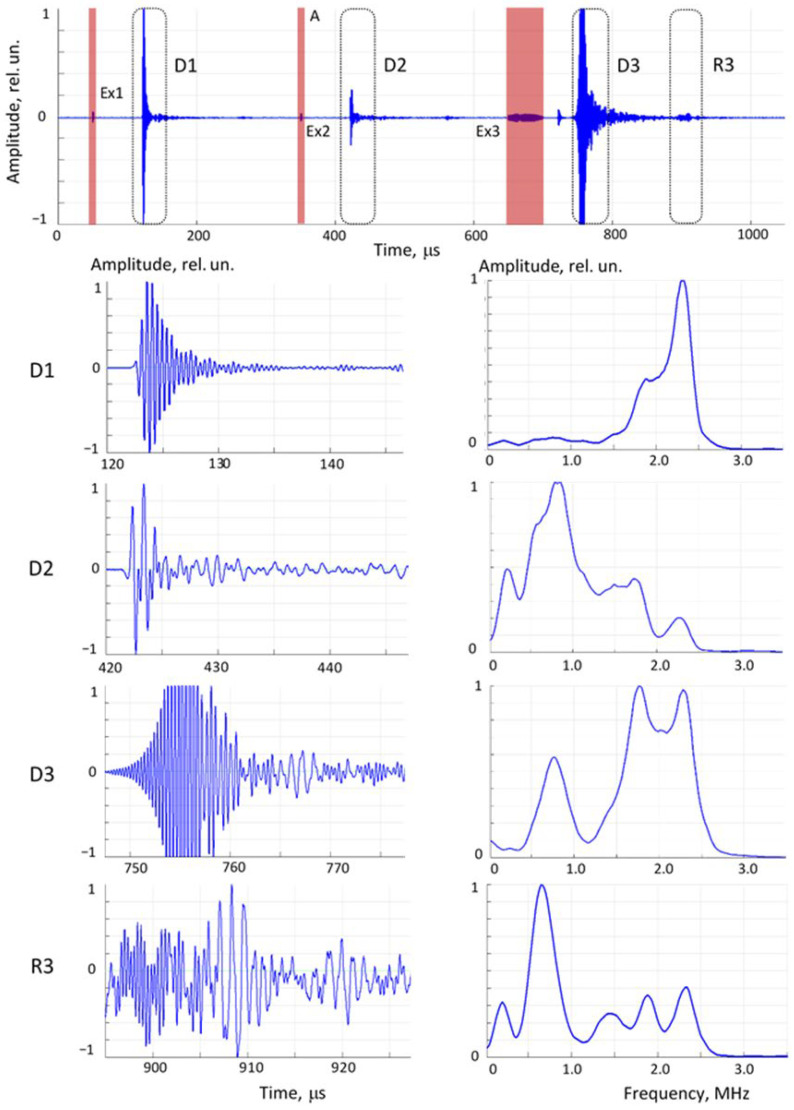
Train of ultrasonic signals acquired in one run (**upper** chart), expanded signal fragments in the time domain within dashed areas (**left** column charts), and their spectra in the frequency domain (**right** column charts). D1 and D2—direct propagation signals at 2.5 MHz (Ex1) and 0.8 MHz (Ex2) excitation; D3—direct propagation signal excited by frequency sweep (Ex3); R3—reflected frequency sweep signal. Red strips denote time of excitation.

The ultrasonic signals were acquired as a signal train containing three separate waveforms per pass: excitations at carrier frequencies of 0.8 MHz and 2.2 MHz and a frequency sweep in the range 0.5 to 2.5 MHz. The sequence of received direct propagation signals, their expanded fragments, and the corresponding spectra after fast Fourier transform processing are illustrated in Figure 5. The frequencies of 0.8 and 2.2 MHz were the resonant frequencies of the disk-type transducers used. The excitation waveforms were tone bursts of two-period sinusoids at these frequencies, enveloped by a half-period sine function. The sweep waveform contained both low-frequency components (below 1 MHz) and high-frequency components (around 2 MHz). The sweep waveform was strong enough to record the second reflection, that is, the ultrasonic signal that had passed through the phantom three times, which was also used in subsequent data processing.

Each phantom was measured 15 times, resulting in 375 individual signal sets (signal trains). The acoustic path length was measured simultaneously using a digital caliper with an accuracy of 0.1 mm.

### 2.3. Determination of Ultrasonic Parameters as Evaluation Criteria for Pattern Recognition and Neural Network Analysis

After considering a number of physical parameters of wave propagation in the phantoms, their variability, and sensitivity to the factors of interest (SAT and IMAT), the evaluation criteria (Cr) were selected. These included ultrasound velocity and parameters related to changes in signal intensity as the wave passed through the phantom at different frequencies (direct propagation and third pass).

Six evaluation criteria were selected, each with different trends and quantitative dependence on SAT and IMAT:

Cr1—ultrasound velocity. Ultrasound velocity C (m/s) was calculated as

C = L/(t − Δt),(1)

where L is the distance between the emitter and receiver, t is the ultrasound arrival time measured by the first zero-crossing as the signal reference point, and Δt is the transducer’s time offset.

Cr2 and Cr3 represent the ultrasound attenuation at frequencies 0.8 and 2.2 MHz, α_0.8MHz_ and α_2.2MHz_, correspondingly.

Ultrasound attenuation at a certain frequency α (dB/cm) was calculated as

α = (20log(I_0_/I_1_))/L, (2)

where I_0_ is the signal intensity or the integral of the signal amplitudes at zero distance; I_1_ is the same at distance L. The integrals I were calculated as the sum of amplitude values S_n_ in the corresponding time windows:

(3) $$I=\sum_{n_{s}}^{n_{e}}\left|S_{n}\right|,$$

where n_s_ is the start index, n_e_ is the end index, and n is the number of signal discretization points.

Cr4—ratio of attenuation values at 0.8 and 2.2 MHz, determined as α_0.8MHz_/α_2.2MHz_;

Cr5—intensity of sweep signal determined as the integral of signal amplitudes in the fixed-length time window;

Cr6—ratio of signal intensities between the sweep signal in direct pass and its second reflection (triple pass through the phantom) (Figure 5).

### 2.4. Methodology of Recognition and Data Structure

Evaluation of IMAT and SAT as factors of interest in the phantoms was based on analysis of the initial data, combined with the principles of pattern recognition and artificial neural networks. The methodology consisted of three main stages:

- Construction and analysis of patterns of evaluation criterion values (Cr) as functions of IMAT and SAT values;
- Application of pattern recognition methods to analyze IMAT and SAT, using decision rules constructed from a training dataset and then applied to test objects;
- Application of artificial neural networks to analyze IMAT and SAT, using training and test datasets with ANN-based analysis of the test objects.

**Figure 6:**
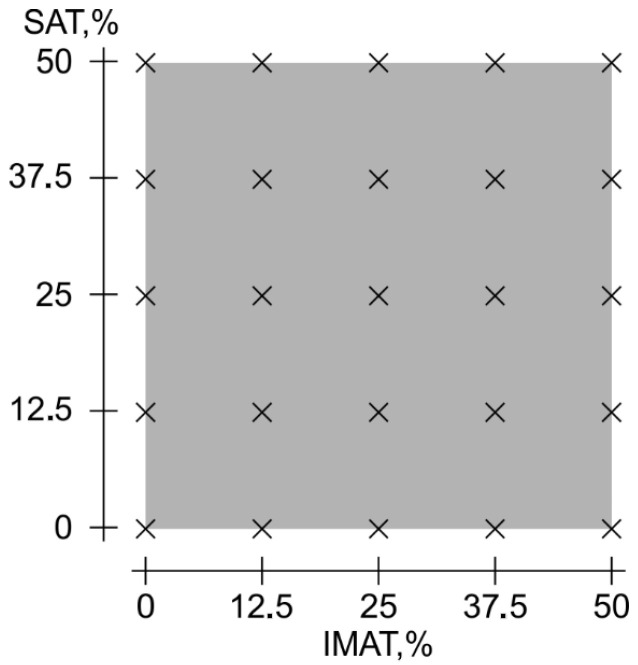
Object (phantoms) grid according to simulated gradations of SAT and IMAT.

The source data used in this work formed an orthogonal grid of objects with uniform gradations of IMAT and SAT values ranging from 0 to 50% in steps of 12.5% (Figure 6).

Each object was described by a set of 15 complex signals, or signal trains, as illustrated in Figure 6. These signals were repeated measurements with the transducers removed and acoustic contact restored between measurements. For each signal, six evaluation criteria, Cr1 to Cr6, were calculated for the two variable factors of interest, SAT and IMAT. For a single signal, the record structure was as follows:

|Signal number|IMAT|SAT|Cr1|Cr2|Cr3|Cr4|Cr5|Cr6|.

Custom software was developed using C++20 software for both feature extraction and pattern recognition analysis. The same software environment was used to visualize and inspect the raw ultrasound signals prior to selecting the features for subsequent processing. The graphical user interface was implemented using Qt 6.7, and geometric computations were performed with the CGAL 6.1 library.

### 2.5. Recognition Based on Pattern Recognition Using Decision Rules

After calculating the evaluation criteria, Cr1 to Cr6, for each signal, the maximum and minimum values of each criterion were determined for each object. These values were used to construct a three-dimensional volume, in which the upper boundary surface was a piecewise linear interpolation function representing the maximum parameter values, and the lower boundary surface was a piecewise linear interpolation function representing the minimum parameter values. To increase the number of data points in this volume, the upper and lower surfaces were further interpolated using Akima interpolation splines, and three new interpolated points were inserted between each pair of original data points. This increased the number of points from 5 to 17 on each axis.

To evaluate a new object with unknown SAT and IMAT values, the same evaluation criteria, Cr1 to Cr6, were calculated from the received signal train. For each criterion, the region of intersection between the calculated criterion value and the corresponding three-dimensional minimum–maximum volume derived from the training set was constructed. This region could consist of one or more intersection areas. Each such intersection region represented a set of possible SAT and IMAT values in a two-dimensional space. To obtain the final estimate, the intersection of all six regions across the six evaluation criteria was determined. The final answer was then localized as the center of mass of the resulting intersection area, with coordinates in the SAT-IMAT plane.

**Figure 7:**
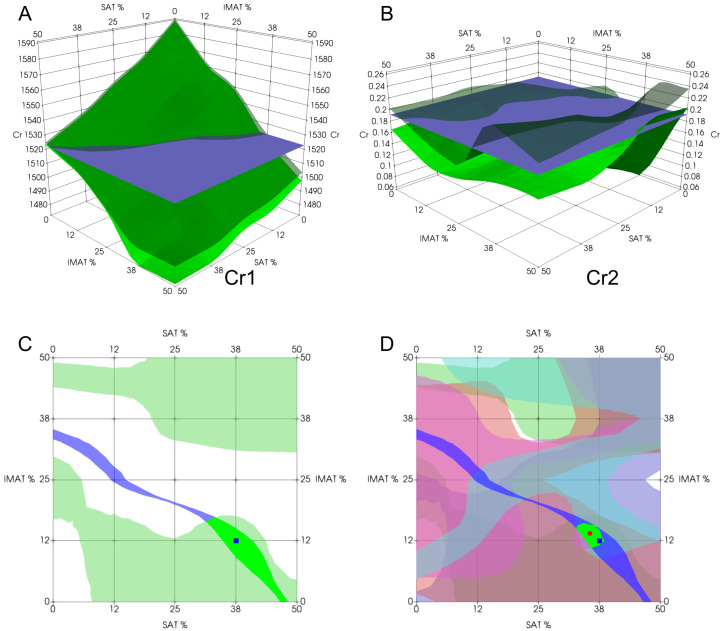
Recognition procedure using decision rules: (**A**)—determination of intersection of one criterion, Cr1, of a test object with three-dimensional minimum–maximum volume of Cr1 determined by training set; (**B**)—the same as (**A**) for Cr2; (**C**)—two-dimensional area of possible answers by combination of criteria Cr1 and Cr2; (**D**)—final answer as intersection area of all criteria Cr1–Cr6. Blue box denotes projected value; red dot denotes estimated value.

The recognition procedure is illustrated in Figure 7. Figure 7A–C show the stepwise application of two decision rules constructed from evaluation criteria Cr1 and Cr2. Figure 7A,B present the intersection between the test object’s feature value (represented by a horizontal plane at the measured criterion value) and the corresponding minimum–maximum decision-rule surfaces. These intersections define two SAT/IMAT segments, which are displayed in different colors in Figure 7C. Their overlap yields a refined sub-segment, thereby narrowing the feasible decision region for SAT and IMAT. For comparison with the known a priori SAT and IMAT values of the test object, Figure 7C also marks the true point, which lies on the same sub-segment—demonstrating the approximate correctness of the assessment even when only two rules are used. Figure 7D shows the final decision obtained by applying all six decision rules in the same manner. The resulting sub-segment becomes substantially smaller, further constraining the solution domain and improving estimation accuracy. In Figure 7D, the blue dot represents the known a priori SAT/IMAT values, while the red dot indicates the estimated values computed as the center of mass of the final sub-segment. This displacement reflects the estimation error in the SAT–IMAT coordinate space.

### 2.6. Recognition Based on Artificial Neural Network

Although LSTM networks are primarily designed for sequential data, in this study, the architecture was applied in a simplified form to process feature vectors derived from ultrasound measurements. Each phantom measurement was represented by six extracted parameters (Cr1–Cr6), forming a single feature vector per sample. In this configuration, the LSTM operates on sequences of length one, which preserves the stable gradient flow and robust training dynamics characteristic of recurrent architectures, even though no temporal dependencies are present.

This setup allows the network to capture nonlinear relationships between the extracted ultrasonic features and the target SAT/IMAT values. The methodology is consistent and reproducible, as all samples undergo identical preprocessing and feature extraction prior to neural network training.

The Long Short-Term Memory (LSTM) method was used as the main neural network method in the experiment. LSTM is a specialized recurrent neural network architecture designed for processing and analyzing data with long-term temporal dependencies. It addresses classical recurrent network issues, such as vanishing and exploding gradients, and enables reliable learning from sequential data. This is achieved through a specific structure that includes memory cells and three main gates: the forget gate, the input gate, and the output gate.

**Figure 8:**
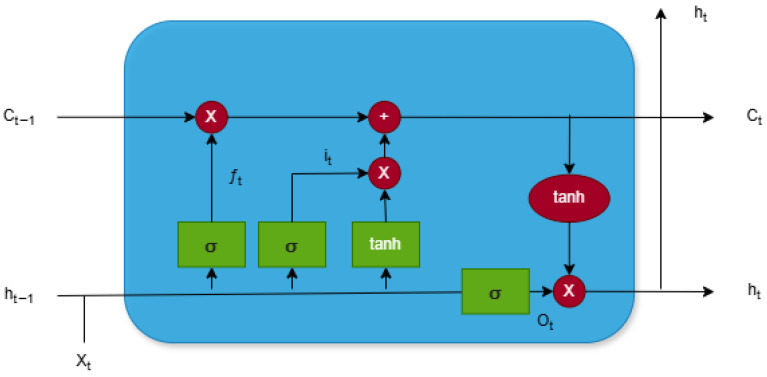
Structure of LSTM cell. X_t_—input vector at time step t; h_t_—hidden state at time t; C_t_—cell state at time t; h_t−1_, C_t−1_—states transferred from the previous time step; i_t_—input gate; f_t_—forget gate; O_t_—output gate; σ—sigmoid activation function; tanh—hyperbolic tangent activation function.

The structure of the LSTM cell is illustrated in Figure 8. The meaning of elements in the structure of an LSTM cell is as follows:

X_t_—input vector at time step t (in this study, a vector of six extracted ultrasound parameters);

h_t_—hidden state at time t, containing the short-term encoded representation;

C_t_—cell state at time t, responsible for preserving long-term information;

h_t−1_, C_t−1_—states transferred from the previous time step;

i_t_—input gate controlling which new information is added to the cell state;

f_t_—forget gate regulating which part of the previous cell state is preserved;

O_t_—output gate determining which portion of the updated cell state contributes to the hidden state;

σ—sigmoid activation function used inside all three gates (forget, input, output) mapping values to the range [0, 1], enabling the gates to selectively pass or block information;

tanh—hyperbolic tangent activation function applied to candidate cell-state values and to the updated cell state before generating the hidden state, mapping values to the range [−1, 1], helping the network model nonlinear relationships and maintain stable gradients.

The model utilized a hyperbolic tangent function (tanh). The hyperbolic tangent function is a commonly used activation function in LSTM networks. This function maps input values in range [−1, 1], making it useful for regulating the flow of information within the network.

(4) $$tanh(x)=\frac{e^{x}-e^{-x}}{e^{x}+e^{-x}},$$

where x is the input value; e is Euler’s number.

Tanh is zero-centered, allowing both positive and negative activation. This property helps in preserving the balance of information during training, reducing bias accumulation.

Validation loss (val_loss), a key metric, was used to evaluate the performance of LSTM model during training. This metric represents the error calculated on the validation dataset, which consists of data that the model has not seen during training. Validation loss is computed using the same loss function as the training loss (Mean Squared Error).

Mean Squared Error (MSE) measures average squared difference between the predicted values and actual target values.

(5) $$MSE=\frac{1}{n}\sum_{i=1}^{N}{\left(y_{i}-{ý}_{i}\right)}^{2},$$

where N is the total number of samples; $y_{i}$ is the actual value of the i-th sample; ${ý}_{i}$ is the predicted value for the i-th sample.

Since error is squared, MSE penalizes larger deviations more heavily than smaller ones, making it sensitive to outliers. A lower MSE indicates better model performance, as it means predictions are closer to the true value.

The R squared score is a statistical metric used to evaluate the performance of regression models. It measures how well a model’s predictions approximate actual values by comparing explained variance to the total variance in data.

(6) $$R^{2}=1-\frac{\sum_{i=1}^{N}{\left(y_{i}-{ý}_{i}\right)}^{2}}{\sum_{i=1}^{N}{\left(y_{i}-ÿ\right)}^{2}},$$

where $y_{i}$—actual value of i-th sample; ${ý}_{i}$—predicted value of i-th sample; ÿ—mean of all actual values; N—total number of samples.

R squared score ranges from −∞ to 1. High R squared score indicates that the model better explains the variance in the target variable. However, a high R squared score does not mean accurate predictions.

All neural network computations were performed using the Python v3.12 programming environment, together with widely adopted scientific and machine learning libraries. Model development and training were carried out using TensorFlow v2.16.1 and Keras v2.15. Data preprocessing, feature handling, and numerical operations were performed using NumPy v2.0, SciPy v1.12, and Pandas v2.2.1, while model evaluation and data partitioning procedures were implemented with Scikit-learn. Training was accelerated on an NVIDIA GPU using the CUDA v9.0 and cuDNN v9.1 libraries, which provided optimized parallel computation for deep learning workloads. The development environment was managed through Microsoft Visual Studio v2022. This computational setup ensured efficient model training and a reproducible workflow for neural network experiments.

## 3. Results and Discussion

### 3.1. Experimental Decision Rules

**Figure 9:**
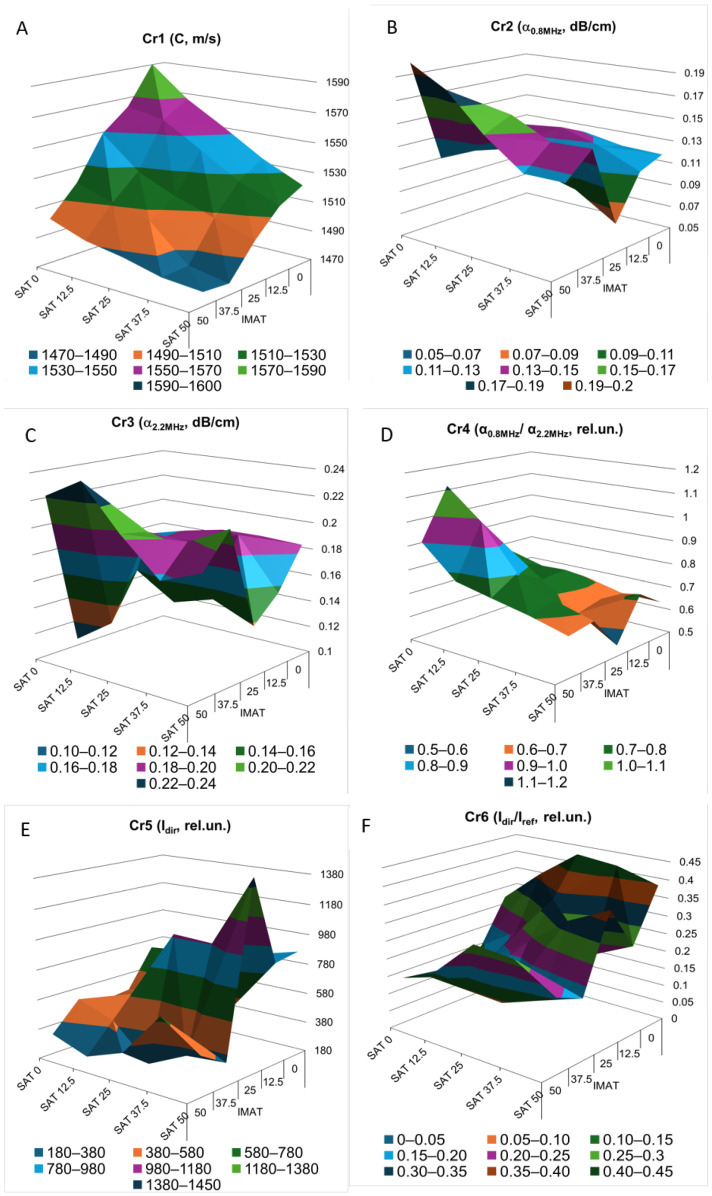
Experimental decision rules constructed for evaluation criteria Cr1 to Cr6 (**A**–**F**) in a three-dimensional domain as piecewise linear functions of SAT and IMAT. Explanations in text.

The three-dimensional images of the decision rules constructed for criteria Cr1 to Cr6 (Figure 9) as piecewise linear functions of SAT and IMAT visualize multidirectional changes in these criteria in SAT-IMAT coordinates. The complex appearance of the graphs reflects the complex influence of SAT and IMAT on these criteria, which appears as multidirectional trends and local extremes in different regions of the SAT-IMAT space.

The main exception is the decision rule for Cr1, which represents ultrasound velocity (Figure 9A). This criterion shows a linear, monotonic decrease along both the SAT and IMAT axes, resulting in a smooth sloping surface close to a plane. The behavior of decision rules for Cr2 to Cr6 (Figure 9B–F) is more difficult to interpret in physical terms without detailed physical and mathematical simulations of the mutual influence of SAT and IMAT on ultrasonic attenuation. An increase in IMAT introduces a scattering effect and causes attenuation of forward ultrasound propagation, while echogenicity in B-mode imaging increases [21]. The presence of two homogeneous SAT layers on both sides partly weakens this effect.

In general, trends were observed toward increasing ultrasound attenuation (Cr2, Cr3, Cr5) and increasing frequency-dependent slope of attenuation (Cr4) with increasing SAT and IMAT, particularly with increasing IMAT due to scattering. Consequently, the ratio of direct signal propagation to its third pass after double reflection (Cr6) decreased. These conclusions, obtained in tissue phantoms, are consistent with the general observation that ultrasound attenuation in lean muscle is approximately half that in fat [23,24]. However, the combined effect of different ratios of homogeneous fat (SAT) and dispersed fat (IMAT) makes the relationships quite complex, as reflected in the complicated shapes of the decision rule surfaces for Cr2-Cr6. In addition, the interfaces between the SAT layers and the hypothetical muscle containing IMAT inclusions make their own reflective contribution. Because a detailed analysis of the physical causes of the complex SAT-IMAT effects was outside the scope of this study, the experimental data were treated as phenomenological, as obtained in the experiment.

**Figure 10:**
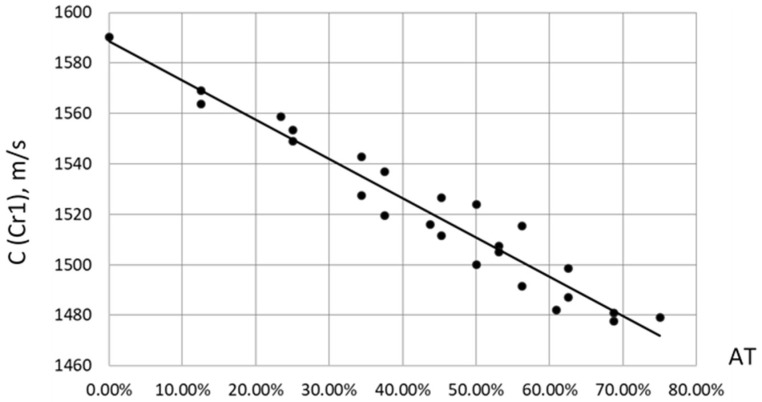
Empirical correlations between ultrasound velocity C and total fat content AT = SAT + IMAT in muscle phantoms.

For ultrasound velocity (Cr1), when correlating velocity C with the total adipose tissue content, defined as AT = SAT + IMAT (%), a clear linear dependence was observed (Figure 10), in the form C = 1589 − 155.9*AT (m/s). The Pearson linear correlation coefficient was *p* = −0.97. In this case, AT varied from zero to 75% at maximum SAT = 50% and IMAT = 50%, because SAT was defined relative to the entire volume of the phantom and IMAT relative only to its muscle component. The slope of C versus AT corresponded to a decline of 1.57 m/s in velocity for each 1% increase in total adipose tissue. This finding closely agrees with results obtained for ultrasound velocity in human lower limb muscle as a function of fat content [39,40].

To assess how much each criterion varied between objects compared with the same within objects and to quantify its discrimination ability, individuality indices (II) and their reciprocals (RII) were calculated. These and other statistical input data are summarized in Table 1. As is generally accepted [41,42], a parameter shows low individuality, or poor discrimination, when II < 0.6 and population-based reference intervals are considered non-informative. When II > 1.4, the parameter exhibits high individuality, i.e., its individual values vary substantially between objects. Conversely, a higher RII is an index of heterogeneity and indicates stronger discriminatory power between objects [41,43]. The obtained data showed the highest RII for Cr1, ultrasound velocity, which was many times higher than for the other criteria. This indicates that ultrasound velocity has much greater discriminatory power compared to criteria based on ultrasound signal intensity. Thus, Cr1 should be considered the primary criterion reflecting the combined influence of SAT and IMAT, whereas the other criteria have a complementary role in discriminating between SAT and IMAT. Because each of the other criteria, Cr2–Cr6, has higher individuality, and thus lower discriminative power, relative to Cr1, their combined use and partial overlap may help improve the resolution of SAT and IMAT.

### 3.2. Evaluation Experiment

In this study, the effectiveness of the proposed evaluation methods was tested experimentally by splitting the scan data in five different ways.

In splits one to three, the data were divided by index. That is, 3 signals with identical indices from the 15 signals in total (repeated measurements with contact reestablished) for each of the 25 objects were used for testing. In splits four and five, three randomly selected signals from each object were used for the test set, and the remaining signals were used for the training set.

The resulting database was used to conduct five similar experiments. In each experiment, the corresponding data split was analyzed using both the decision rule approach and the artificial neural network approach (LSTM).

### 3.3. Recognition Results

**Figure 11:**
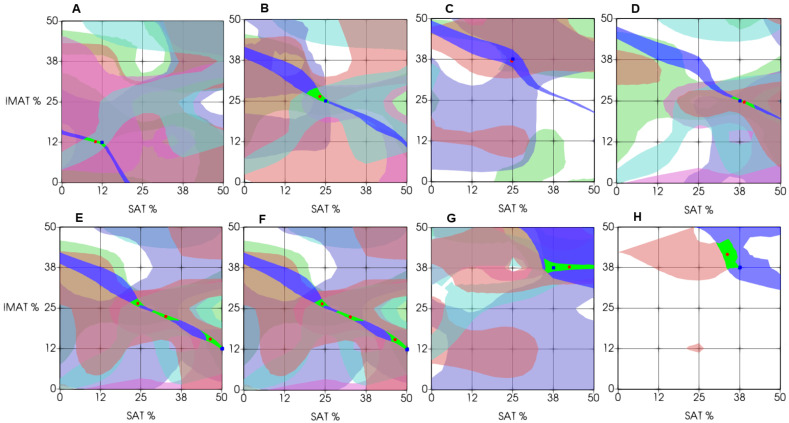
Examples of differential estimation of SAT and IMAT using decision rules. (Green area—intersection of the maximum number of decision rules; blue square—predicted value; and red circle—experimental estimation.) The top and bottom rows illustrate four examples of precise evaluation and four examples of dispersed evaluation, respectively: (**A**–**D**)—examples of high estimation accuracy; (**E**,**F**)—examples of uncertainty due to multiple intersection areas; (**G**)—example of partially out-of-bounds intersection area; (**H**)—example of lack of intersecting decision rules.

Figure 11 demonstrates the characteristic results of experimental estimation of IMAT and SAT in eight test objects using the proposed decision rules approach. These examples are chosen to show cases of varying degrees of estimation accuracy. The examples are chosen to illustrate different degrees of estimation accuracy. In general, the estimation result (the green intersection segment of all six decision rules, centered on the red dot) shows diagnostically acceptable accuracy compared with the a priori projected values of the test object (blue point). As can be seen in Figure 11, in most cases, the estimate was accurate within the accuracy of phantom fabrication (panels A–D). However, decision rules sometimes produced multiple intersection segments, leading to multiple possible answers (panels E and F). In such cases, an average value can be calculated across all possible answers, but this does not necessarily yield an exact match with the projected value. Furthermore, when using a decision rule-based approach, situations may occur in which some of the decision rules do not intersect or lie partially outside the recognition area, and no intersection segment is formed. Panels G and H illustrate such cases. In these situations, the decision segment is derived from the intersection of fewer than six criteria.

Table 2 summarizes the statistical evaluation of SAT and IMAT estimation accuracy obtained using the pattern recognition approach across five data splits. The accuracy metrics include the correlation coefficient (R), the coefficient of determination (R^2^), the sum of squared errors (SSE), and the standard error of estimate (SEE). The average SEE values are 6.3% for SAT and 5.5% for IMAT, indicating that the overall estimation error is approximately 6%. These results show that the method achieves an acceptable level of precision for differentiating adipose tissue components under controlled experimental conditions.

### 3.4. Artificial Neural Network Analysis

Neural network experiments were performed using the same dataset and data partitions as in the pattern recognition experiments, ensuring identical training and testing conditions. For each of the five data splits, an LSTM network was trained independently to estimate IMAT and SAT parameters from the corresponding sequences of ultrasound signals.

Before training, all input features were normalized to a common numerical range of 0–1 to ensure that each parameter contributed proportionally during optimization. Normalization factors were computed exclusively from the training subset to avoid information leakage, and the same scaling was subsequently applied to the validation and test sets.

For model evaluation, the dataset was partitioned into independent subsets: 20% of the samples were reserved as a held-out test set, and the remaining 80% were split into training and validation sets using a fixed random seed to ensure reproducibility. The validation set was used solely for monitoring convergence and applying early stopping.

**Figure 12:**
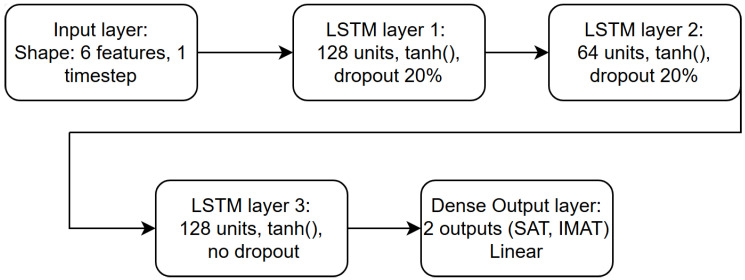
Structure of the LSTM network.

The LSTM network comprised three recurrent layers with 128, 64, and 128 units, respectively, with a dropout rate of 0.2 between layers to improve generalization. Training was performed using the Adam optimizer with a batch size of 16 for up to 1500 epochs. Early stopping with a patience of 150 epochs was used to prevent overfitting and to retain the model achieving the lowest validation loss. Mean Squared Error (MSE) served as both the loss function and the primary performance metric. Figure 12 illustrates the LSTM architecture used in this study.

The Mean Squared Error (MSE) was used as the loss function, and both MSE and the coefficient of determination (R^2^) were monitored during training to assess model performance. These metrics were calculated after each epoch for both training and validation subsets to ensure stability and convergence of the learning process. The training dynamics were generally consistent across all data splits, with rapid stabilization in the early epochs and smooth convergence toward the optimal solution, without evidence of overfitting.

After training, each optimized model was applied to the corresponding test subset to obtain predicted IMAT and SAT values. The predicted values were collected for further analysis and comparison with reference parameters.

This experimental design allowed for a reproducible and balanced assessment of the neural network’s generalization ability under conditions identical to those used for the pattern recognition approach.

### 3.5. Comparative Assessment of Recognition Accuracy by Pattern Recognition and Artificial Neural Network Analysis

The average accuracy scores and their deviations for each data split, obtained with the pattern recognition and decision rule (PR/DR) approach and with the LSTM neural network (ANN/LSTM), are summarized in Table 3. The results show that estimation accuracy varies slightly depending on how the ultrasonic data are split.

On average, when using decision rules (PR/DR), the IMAT estimation error across different splits ranged from about 3 to 4%, with an average value across all objects of about 3%. The average SAT estimation error was slightly higher, ranging from about 4 to 5%, with an average value of 4.6% across all data. Even in a clinical setting, such an error would provide an assessment accuracy comparable with that achieved by imaging modalities.

When using ANN/LSTM, the average IMAT estimation error across different splits ranged from 0.4 to 1.2%, with an average value of about 0.7% across all data. The SAT estimation error falls within similar ranges. Therefore, the use of neural networks facilitates a substantial reduction in the average estimation error compared with traditional pattern recognition methods, while maintaining robustness with respect to data partitioning. It must be acknowledged, however, that these results were obtained under ideal laboratory conditions using geometrically homogeneous objects.

**Figure 13:**
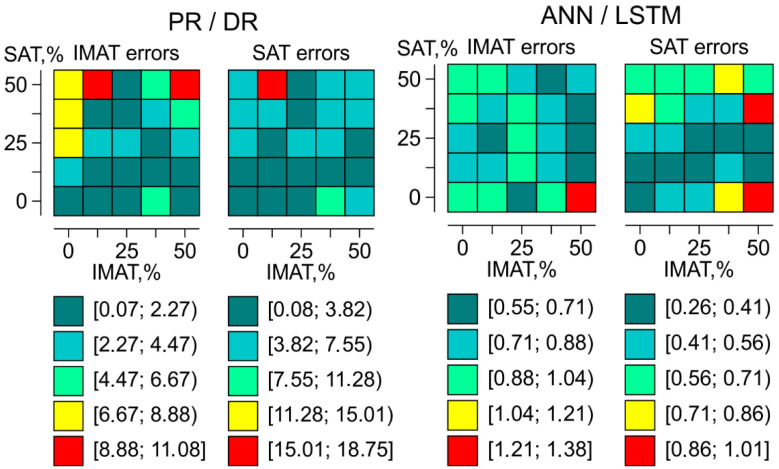
Topology of SAT and IMAT evaluation errors across the SAT-IMAT distribution field using PR/DR and ANN/LSTM.

The topology of SAT and IMAT evaluation errors across the SAT-IMAT parameter field for PR/DR and ANN/LSTM is presented in Figure 13. Each cell of the 5 × 5 matrix corresponds to a phantom with a specific SAT and IMAT content and displays the magnitude of SAT or IMAT recognition error for that object obtained by the respective method.

For the PR/DR method, areas of low error are observed at low and moderate IMAT and SAT values. As SAT and IMAT increase, the errors increase, reaching the highest values in the upper part of the diagram, that is, at high SAT. This suggests that the PR/DR method provides more accurate predictions of IMAT at low and moderate SAT. Consequently, PR/DR may be better suited for assessing intermuscular fat in lean and non-obese patients, whereas high levels of obesity may prevent accurate differentiation of IMAT from total limb fat (SAT plus IMAT).

In contrast, the error values obtained using ANN/LSTM are lower by approximately one order of magnitude and more evenly distributed across the entire parameter range than those produced by PR/DR. Most of the parameter domain shows low errors, from about 0.26 to 1.38%. Small local increases in error are observed mainly at high IMAT and SAT values, but even these remain significantly lower than the corresponding errors obtained with PR/DR. These findings show that neural network analysis can provide substantially higher accuracy than the pattern recognition method. In practice, however, a considerably larger training database may be required to account for the heterogeneity of individual anatomical features in humans. The advantage of the PR/DR method is that it can operate with a comparatively limited database.

### 3.6. Limitations of the Study and Prospects for Further Research

This study represents an initial demonstration that SAT and IMAT in human limbs can be differentiated using a simple single-channel through-transmission ultrasound system combined with AI analysis. Using idealized phantoms with linearly varying SAT and IMAT, we achieved the primary goal of establishing feasibility.

The approach is empirical and phenomenological. Several evaluation criteria beyond ultrasound velocity show complex and nonlinear behavior with local extrema across the SAT–IMAT space (Figure 9). Such relationships are inherently challenging to interpret unequivocally and highlight the need for complementary theoretical modeling. Mathematical simulations that systematically vary SAT and IMAT could clarify their combined influence on specific aspects of ultrasound propagation. These directions constitute important avenues for future research.

Quantitative ultrasound of muscle is strongly affected by SAT and IMAT [44], yet simulations of their joint effects remain limited. Foundational work with finite-difference time-domain (FDTD) simulations demonstrated that soft-tissue heterogeneities, including skin, SAT, muscle, and connective tissue, induce substantial amplitude and phase distortions [45]. More recent modeling frameworks built on pseudo-spectral k-space frameworks (such as k-Wave) enable full-wave modeling of heterogeneous, absorbing media [46], and mixed-domain approaches have been validated for tissues with spatially varying acoustic properties [47]. These studies collectively indicate that SAT and IMAT must be modeled together, as their effects on attenuation, scattering, and phase cannot be linearly superimposed.

High-resolution MRI distinguishes SAT, IMAT, and intramyocellular fat and provides PDFF-based acoustic maps for simulation [48]. Automated MRI segmentation and CT-based adipose quantification are already used to assign spatially varying properties in ultrasound models [45]. MRI–ultrasound comparisons confirm that image-derived fat fraction correlates with echo intensity and attenuation [6]. Thus, MRI and CT datasets can supply ground-truth maps of SAT and IMAT for wave modeling and for training AI systems to discriminate these depots in future clinical studies using the method proposed here.

## 4. Conclusions

This model-based study confirms the fundamental feasibility of distinguishing between subcutaneous adipose tissue (SAT) and intermuscular adipose tissue (IMAT) using multifrequency, at least dual frequency, ultrasound signals collected in through-transmission in human limbs, in combination with artificial intelligence-based processing methods.

The applied pattern recognition approach, based on decision rules constructed from ultrasound propagation parameters used as evaluation criteria, demonstrated diagnostically acceptable accuracy in determining SAT and IMAT and shows promise for transfer to in vivo applications. Although the artificial neural network-based approach (ANN/LSTM) achieved recognition accuracy that was approximately an order of magnitude higher, the value of the pattern recognition method lies in its ability to work with a relatively small number of objects in future studies.

The limitations imposed by the model study, such as constant phantom geometry and regular, idealized SAT and IMAT inclusions, may present challenges when extending the mathematical model to human studies. In real subjects, the anatomical complexity and heterogeneity of SAT and IMAT distribution may require additional modeling and correction strategies. The use of imaging modalities such as MRI and CT as reference standards could help to refine the model by incorporating additional evaluation criteria with appropriate weighting factors.

In addition, limitations arising from the use of simplified phantoms mean that the present results should be interpreted as a proof of concept. Future work in human subjects will need to account for anatomical variability, tissue anisotropy, and complex boundary conditions. Imaging techniques can assist in this process by providing ground truth references and supporting the integration of further parameters into the recognition framework.

## Figures and Tables

**Table 1:** Statistical measures of evaluation criteria.

Criterion	Cr1	Cr2	Cr3	Cr4	Cr5	Cr6
Measurement unit	m/s	dB/cm	dB/cm	rel.un.	rel.un.	rel.un.
Mean for the entire dataset (M)	1520.0	0.131	0.745	0.177	0.235	594.8
Standard deviation for the entire dataset (SD.m)	±30.3	±0.028	±0.139	±0.033	±0.113	±307.1
Coefficient of variation for the entire dataset (CV.m)	2.0%	21.4%	18.7%	18.4%	48.2%	51.6%
Mean standard deviation for the individual objects (SD.o)	±1.31	±0.012	±0.059	±0.013	±0.034	±102.8
Mean coefficient of variation for individual objects (CV.o)	0.1%	9.4%	7.9%	7.2%	14.6%	17.3%
Index of individuality (II = CV.o/CV.m)	0.04	0.44	0.42	0.39	0.30	0.33
Reciprocal of II (RII = 1/II)	23.2	2.3	2.4	2.6	3.3	3.0

**Table 2:** Statistical errors in SAT and IMAT assessment in the experiment, depending on data splits.

Data Splitting	SAT	IMAT
Split 1	R: 0.961; R^2^: 0.924 SSE: 595.7; SEE: 5.09	R: 0.912; R^2^: 0.832 SSE: 1313.2; SEE: 7.56
Split 2	R: 0.935; R^2^: 0.874 SSE: 982.8; SEE: 6.54	R: 0.966; R^2^: 0.937 SSE: 525.8; SEE: 4.78
Split 3	R: 0.949; R^2^: 0.901 SSE: 772.3; SEE: 5.79	R: 0.973; R^2^: 0.948 SSE: 408.5; SEE: 4.21
Split 4	R: 0.957; R^2^: 0.916 SSE: 653.8; SEE: 5.33	R: 0.963; R^2^: 0.929 SSE: 565.1; SEE: 4.96
Split 5	R: 0.870; R^2^: 0.757 SSE: 1743.3; SEE: 8.90	R: 0.942; R^2^: 0.888 SSE: 805.9; SEE: 6.05
Average error across all splits	R: 0.934; R^2^: 0.874 SSE: 949.6; SEE: 6.33	R: 0.951; R^2^: 0.905 SSE: 723.7; SEE: 5.5122

**Table 3:** Comparison of mean evaluation errors (percentage) for SAT and IMAT using pattern recognition PR/DR and artificial neural networks ANN/LSTM.

Data Splitting	PR/DR		ANN/LSTM	
	IMAT	SAT	IMAT	SAT
Split 1	3.88 ± 3.61	5.25 ± 5.20	0.74 ± 0.32	0.37 ± 0.26
Split 2	3.06 ± 3.80	4.52 ± 4.58	0.40 ± 0.30	0.34 ± 0.23
Split 3	2.97 ± 3.15	4.29 ± 3.79	1.20 ± 0.62	0.45 ± 0.43
Split 4	3.36 ± 3.76	3.84 ± 3.80	0.48 ± 0.33	0.65 ± 0.34
Split 5	3.75 ± 4.94	5.08 ± 7.34	0.46 ± 0.28	0.41 ± 0.27
Average error across all splits	3.14 ± 3.85	4.60 ± 5.06	0.65 ± 0.49	0.41 ± 0.27

## Data Availability

The original contributions of the study are included in the article; further inquiries can be directed to the corresponding author.
